# Vital Statistics of Marshall County, Ky.

**Published:** 1852-03

**Authors:** George W. Irvin

**Affiliations:** Benton, Ky.


Art. II.—*Vital Statistics of Marshall County, Ky.* By George W. Irvin, M. D., of Benton, Ky.

Marshall county is situated in that portion of the State of Kentucky lying west of the Tennessee river, and familiarly known as Jackson's purchase. It is washed on the north-east by the Tennessee river, which is the boundary line between it and the counties of Trigg and Livingston. On the south it joins Calloway county, and on the west Graves and McCracken. It is lower than the country east and south, and slopes gently towards the north-west.

There has been no geological survey of the county. The surface and sub-soil are clay over a sandy foundation. Those best informed on the subject are of opinion that the formation belongs to the tertiary. The Tennessee river on the north-east separates this from the limestone formation, and there is but a single instance of the existence of carboniferous limestone in the country, which is near the Tennessee river, where it occurs in a single knob. The rocks (of which there are but few) are conglomerate, composed principally of quartz and the red and black oxides of iron, and sufficiently hard for millstones. The pebbles composing this rock exhibit traces of organic remains, and from the traces of coral sometimes seen, it appears very probable that those remains are referable to a marine, rather than to a fluviatile origin. They consist of shells so broken and rounded as to be difficult to classify. The conglomerate rocks form in some places a crust at the depth of ten or twelve feet below the surface, not however of sufficient thickness to require blasting. Sandstone is said to exist here in some instances and probably lies beneath the region of coal (of which none is found here), which circumstance seems to indicate that this is of an older formation than the country east of the Tennessee river. The Tennessee river runs close to the edge of the limestone formation, having in but a single instance within my knowledge separated any portion so as to have left it on the west side of the river. Iron and its oxides abound here. Nodular masses of argillaceous iron ore are to be found, as also sulphur in combination with iron, and some sulphurets of lead. It is probable that at the time the marine formations of the carboniferous regions of Kentucky and the adjacent States, was taking place, from some circumstance, (as the depth of the sea or the prevalence of hot springs) the same did not take place here; and in the mean time the hot water, impregnated with silex, hardened and silicified whatever marine production came within its influence.

Water is obtained from wells and springs, (mostly from wells) though there is quite a number of excellent springs in the county. Plenty of water may generally be had at the depth of thirty or forty feet below the surface, and when good is usually found in a stratum of white sand. When found in a black clay (as is not unfrequently the case) it is so strongly impregnated with coperas as to be unfit for use. The water is genrally impregnated with iron and its sulphates, sulphuretted hydrogen, &c. Stock water is in many parts of the county very scarce during the dry season of the year.

Clark's river (a small stream) runs through the county in a north-western direction, and nearly parallel with Tennessee river, dividing the county into two nearly equal parts. The banks of this stream (as also those of the Tennessee) are very low, and the bottom lands quite extensive. These bottoms overflow during the rainy season and continue so wet throughout the summer and fall (unless these should be unusually dry) that the roads through them are almost impassable. The bottoms are in many places covered with vegetation; thus there are all the essential conditions for the evolution of malaria during the long continued heat of the summer and fall. On this stream also there are several mill ponds, the vicinity of which is especially pestiferous.

Benton, the county seat of Marshall, is a pretty little village, containing twenty-four families. It was located in 1842, and is situated near the centre of the county, twelve miles west of Tennessee river, and a mile and a half west of Clark's river, on a considerable elevation, and is probably one of the healthiest situations in the county. The houses are of wood with the exception of the court-house, which is a good two story brick building, situated in the centre of a beautiful square.

Owing to the fact that our county has been but recently settled, the improvements are not of the best sort, being intended, (a great many of them) only for temporary use. They are however, already giving way gradually to better buildings, and there are many evidences that we shall ere long rival our neighbors on the east side of the Tennessee river.

I do not, upon the whole, consider the county remarkably unhealthy notwithstanding the small average age of its inhabitants, (as shown by the following tables) which may I think be sufficiently accounted for by the fact of the county's having been but recently settled by emigrants, for it is known that comparatively few aged persons emigrate. Indeed it is a rare thing to see a very aged person here; the adult population are generally men in the prime of life; add to this that there are a great many children here (an evidence of its healthfulness), and it will be seen at once that, though ever so healthy, still on multiplying the combined ages of the inhabitants by their number the quotient must necessarily be small. The malarious fevers are endemic here, but few persons die of them compared to the number attacked. The fatal diseases prevalent here are pneumonia, dysentery, and occasionally, [though I think rarely] typhus and typhoid fevers.

The portions of the county considered to be the most unhealthy, and which I am pretty well assured have been so since I came to the county, in April, 1850, are those lying in the vicinity of the Tennessee and Clark's rivers, together with a small portion of the northern part of the county, lying on Cypress creek, just above where it empties into the Tennessee river. Cypress is a sluggish stream bordered by swamps which, I have been informed, are in some places quite impassable. The reasons of the greater prevalence of disease in these localities, than elsewhere in the county, will readily enough suggest themselves after what has been said descriptive of them.

The past year has been one of unusual sickness here; more so indeed than any year since 1846. The spring was uncommonly wet, and the following summer and fall extremely hot and dry. Scarcely a family in the county escaped more or less sickness, and it was not uncommon to see all the members of a family down at one time with remittent or intermittent fever or dysentery. In the bounds of my practice diseases of the bowels, (either idiopathic or intercurrent) were unusually frequent, rendering necessary great caution in the use of purgative medicines.

There is prevailing here, at present, an epidemic measles of great severity, the convalescence from which is uncommonly slow. The diseases prevailing at present all show a marked tendency to take on a low, asthenic grade of action. Indeed I consider the prevailing epidemic constitution eminently typhoid.

In some places in the county "milk sickness" is so rife as to deter persons from purchasing property in such localities. It seems however to be gradually receding as the county becomes settled, and, I doubt not, will eventually disappear.

There is neither hospital or poor house in the county. There are thirty-two schools, and seven hundred and sixty-two children in attendance, being only about half the children in the county, of whom there are one thousand five hundred and sixty-five.

The entire population of the county is five thousand two hundred and eighty-nine, of which number five thousand and twenty-one are white persons, nineteen are free blacks, and two hundred and forty-nine are slaves. Of the whites, two thousand five hundred and sixty-four are males, and two thousand four hundred and fifty-seven are females. After deducting seven persons whose ages are not given, the remaining five thousand and fourteen afford an average of 16.90. Of the free blacks, eleven are males, and eight are females; and their average is 33.42, which is much more than that of the whites, and is attributable, mainly, to the fact, that many of them have not been freed until unfit for service.

Of the two hundred and forty-nine slaves, one hundred and nine are males, and one hundred and forty are females, with an average age of 17.63—still something over that of the whites, which is probably attributable to the fact, that quite a number of young negroes have been sold and taken away out of the county, while the old servants have been retained.

There were sixty-one deaths in the county during the year ending June 1st, 1850, of which number fifty-nine were white persons, and two negroes. Of these, twenty-nine were under ten years of age at the time of their death; seven between ten and twenty; eight between twenty and thirty; one between thirty and forty; seven between forty and fifty; four between fifty and sixty; one between sixty and seventy; and four between seventy and eighty. Their average age, at the time they died, was 21.81. Of consumption three died; one of remittent fever; one of infantile remittent fever; five of cholera; three of old age; two of apoplexy; eight of croup; two of pneumonia; three of typhus fever; two of inflammation of the bowels; one of constipation; one scalded; two of dropsy; one of small pox; one drowned; one of convulsions; one of pleurisy; one female at the critical period of life; one by falling from a horse; two of intermittent fever; one of fever; one of encephalitis; three of erysipelas; one of an affection of the leg, probably white swelling; and in the cases of the remaining nine, the cause of death was not known.

In February, 1848, the clerk's office with all the records of the county were destroyed by fire, so that I can only give the number of marriages during each month since that time up to January 1st, 1852.

TABLE NO. 1.

*Population.*

| COLOR. | *Males.* | *Females.* | *Total.* | *Av'ge Age.* |
|---|---|---|---|---|
| Whites, | 2,561 | 2,453 | 5,017 | 16.90 |
| " | 3 | 4 | 7 | Unkown. |
| Free Blacks, | 11 | 8 | 19 | 33.42 |
| Slaves, | 109 | 140 | 249 | 17.63 |
| | 2,684 | 2,605 | 5,289 | 17.002 |

### Table No. 2.

*Marriages since February 29th, 1848.*

| Month. | 1848. | 1849. | 1850. | 1851. |
|---|---|---|---|---|
| January, | | 2 | 12 | 3 |
| February, | | 3 | 8 | 7 |
| March, | 7 | 3 | 2 | 4 |
| April, | 3 | 7 | 4 | 4 |
| May, | 6 | 6 | 1 | 4 |
| June, | 4 | 4 | 5 | 5 |
| July, | | 9 | 2 | 5 |
| August, | 4 | 6 | 5 | 1 |
| September, | 4 | 3 | 5 | 2 |
| October, | 6 | 4 | 5 | 6 |
| November, | 5 | 9 | 6 | 10 |
| December, | 5 | 8 | 9 | 2 |

### Table No. 3.

*Number of Deaths during the year ending June 1st, 1850.*

| *Name of Disease.* | *Number.* | *Name of Disease.* | *Number.* |
|---|---|---|---|
| Unknown, | 9 | Convulsions, | 1 |
| Consumption, | 3 | Pleurisy, | 1 |
| Remittent Fever, | 1 | Critical time of Life, | 1 |
| Infantile Remittent, | 1 | Intermittent Fever, | 2 |
| Cholera, | 5 | Fever, | 1 |
| Old age, | 3 | Encephalitis, | 1 |
| Apoplexy, | 2 | Erysipelas, | 3 |
| Croup, | 8 | Of an Affection of the Leg, | 1 |
| Pneumonia, | 2 | Constipation, | 1 |
| Typhus Fever, | 3 | Fall from a Horse, | 1 |
| Diarrhea, | 2 | Drowned, | 1 |
| Puerperal Fever, | 2 | Scalded, | 1 |
| Inflammation of the Bowels, | 2 | | |
| Dropsy, | 2 | Total, | 61 |
| Small Pox, | 1 | | |

Average ages at death, 21.81 years.

Of these persons, fifty-nine were whites, and two were blacks.

TABLE NO. 4.

*Number of Deaths during each Decade of Life.*

| | |
|---|---|
| Under 10 years,............29 | Between 40 and 50 years, ....7 |
| Between 10 and 20,.........7 | " 50 " 60,.........4 |
| " 20 " 30,.........8 | " 60 " 70,.........1 |
| " 30 " 40,.........1 | " 70 " 80,.........4 |

